# A positive mental imagery intervention for targeting suicidal ideation in university students: A pilot study

**DOI:** 10.1002/cpp.2720

**Published:** 2022-02-13

**Authors:** Hayley Knagg, Daniel Pratt, Peter J. Taylor, Jasper Palmier‐Claus

**Affiliations:** ^1^ Centre for New Treatments and Understanding in Mental Health, School of Health Sciences The University of Manchester Manchester UK; ^2^ Greater Manchester Mental Health NHS Foundation Trust Manchester UK; ^3^ Manchester Academic Health Science Centre Manchester UK; ^4^ Spectrum Centre for Mental Health Research, Division of Health Research Lancaster University Lancaster UK; ^5^ Lancashire & South Cumbria NHS Foundation Trust Lancashire UK

**Keywords:** broad‐minded, imagery, student, suicide, therapy

## Abstract

**Objectives:**

Suicide is a major public health concern and is now considered to be the leading cause of death in young people. Suicidal ideation within student populations has recently increased. The Broad‐Minded Affective Coping (BMAC) offers a brief psychological intervention targeting suicidal ideation by enabling access to competing positive emotions and thoughts using guided imagery. Its acceptability and feasibility in student populations are unclear.

**Design:**

A single arm pilot study investigated the feasibility and acceptability of a six‐session BMAC intervention for university students experiencing suicidal ideation.

**Method:**

Recruitment took place from university counselling services. Suicidal ideation and emotional states were assessed at baseline and after 6 and 12 weeks. Participants also completed corresponding sessional measures.

**Results:**

Twelve eligible participants consented to take part with 11 receiving the intervention. Ten participants completed post treatment and follow up assessments. Retention to treatment was high with participants attending an average of 5.2 (87%; SD = 1.54) out of six intervention sessions. There were also good completion rates of the BMAC technique between sessions. Participants reported high levels of satisfaction with the intervention. There was an associated reduction across a range of clinical outcomes, including suicidal ideation, with large effect sizes.

**Discussion:**

This pilot study showed promising results on the feasibility and acceptability of the BMAC intervention in students experiencing suicidal ideation. However, the study had a small sample size and no comparator control group. Further exploration of the BMAC intervention is warranted.

**Key Practitioner Message**

- It was feasible to deliver the Broad‐Minded Affective Coping (BMAC) intervention in university students experiencing suicidal ideation.
- Levels of acceptability were also high.
- Suicidal ideation improved following the BMAC intervention and after 12 weeks.

## 1 INTRODUCTION

Suicide claims over 800,000 lives each year (World Health Organization, 2014), and many of these are young people aged 15–29 (Nock et al., 2013). Since 2010, there has been a rise in rates of suicide amongst young people in England (Bould et al., 2019; Office for National Statistics, 2018) with suicide now being their leading cause of death in England and Wales (Office for National Statistics, 2017). There are growing concerns about the mental health and risk of suicide amongst university students in the United Kingdom and worldwide (Gunnell et al., 2020). Suicidal ideation within student populations is becoming increasingly common (Russell et al., 2019). Gunnell et al. (2020) found that the incidence of suicide in students increased by 15% between 2000 and 2017. Considering that there are higher rates of suicidal ideation relative to suicide attempts (Rogers & Joiner, 2017), it is likely that the issue of suicidal thinking is underestimated in students (Morgan et al., 2019). There continues to be a need for further research and understanding into suicidal ideation within this population.

Suicidal ideation is often a sign that a person is experiencing extreme psychological distress (Garlow et al., 2008) and is considered an important risk factor for future suicidal behaviour and completed suicide (Large et al., 2021; Ribeiro et al., 2017; Simon et al., 2018). Suicidal ideation and/or behaviour for students can be precipitated and exacerbated by a variety of stressors associated with university life, including financial, social, and academic pressures (Auerbach et al., 2016). As a result, university students are vulnerable to psychological distress (Taylor et al., 2013), self‐injury, suicidal ideation, and suicidal behaviour (Garlow et al., 2008; Thorley, 2017).

Support services within universities are well placed to understand the needs of and support students whose lives are often transitory. Given that over 50% of young people in the United Kingdom go into higher education, university‐based interventions have the potential to reach many young people (National Institute for Health Research, 2021). In light of the recent increase in suicide rates in young people, it is important that more targeted prevention interventions are provided for at‐risk students. However, the evidence base within this area is currently limited (Witt et al., 2019). Universities UK and the Office for Students have initiated campaigns and research projects aimed at reducing mental health difficulties and suicide rates in students studying in British universities (Akram et al., 2020). Despite this, student services are continuing to struggle to meet increased demand for support (Mortier et al., 2018; O'Neill et al., 2018). Suicidal ideation, if left untreated, may lead to a number of negative consequences for all involved. Therefore, it is crucial that researchers endeavour to establish early and effective prevention treatment strategies aimed at reducing suicidal ideation in university students (O'Connor & Nock, 2014).

There is a growing body of work suggesting that approaches cultivating positive emotions have an important place within clinical treatment (see Morris et al., 2013) and can buffer against suicide (Rebellon et al., 2000). The broaden and build theory (Fredrickson, 2001) posits that negative emotions restrict attention and cognition to focus on threat, whereas positive emotions enhance attentional focus, encourage exploration and creative processing, and help to build useful skills and psychological resources. It is suggested that focusing on the positives, through the stimulation of positive affect, reduces the influence of threat on attention and information processing (Tarrier et al., 2013). Rojas et al. (2015) found that difficulties experiencing positive affect, and intense negative affect, were correlated with increased suicidality. Differential Activation Theory (DAT; Teasdale, 1988) suggests that there are emotionally activated cognitive networks. For example, during episodes of depression associations are formed between sad mood and a constellation of negative dysfunctional beliefs and cognitive processing biases. With each episode of depression that occurs, the network of these negative cognitions becomes strengthened, elaborated and increasingly accessible. As a result, even relatively small shifts in mood gain the capacity to activate negative thinking patterns (Segal et al., 1996). Whilst originally developed in the context of depression, DAT could be extended to think about emotional distress more generally, and how suicidal ideation could form part of a cognitive network that is triggered at times of distress. This implies that if an individual has had suicidal ideation whilst experiencing a period of distress (e.g. low mood or heightened anxiety), the likelihood that they will experience suicidal ideation again during subsequent episodes is markedly increased (Williams et al., 2006). In a review by Garland et al. (2010), the authors suggest that the experience of positive emotions can counteract the spiral of negative internal states that characterize psychopathology.

The clinical utility of positive emotional experiences inspired the development of the Broad‐Minded Affective Coping (BMAC; Tarrier, 2010; Johnson et al., 2013). The BMAC is a clinical technique that utilizes mental imagery to aid the recall of positive autobiographical memories to elicit positive affect (Panagioti et al., 2012). It has been well documented that mental imagery can elicit strong emotional responses (Holmes et al., 2008; Holmes & Matthews, 2010). The BMAC aims to encourage the person to re‐experience their positive memories and achieve a ‘felt sense’ of being back in the recalled situation. The BMAC then brings the person's attention to the associated positive emotions, as well as eliciting, elaborating, and processing positive personal meaning held by the individual that may counteract negative beliefs (Holden et al., 2017). Tarrier (2010) proposed that this technique offers the opportunity to develop a more balanced perspective by focussing attention on positive experiences. Over time, these positive reflections can then be integrated into a person's self‐concept and worldview, thus bringing about change through the development of positive schemas. The BMAC may also help to build skills in emotional regulation, increasing an individual's awareness of how cognition and attention affect emotions, improving attentional control, facilitating a sense of hope (Johnson et al., 2013) and connectedness (Holden et al., 2017), which are key protective factors against escalating suicidal ideation, particularly amongst those experiencing pain and hopelessness (Klonsky & May, 2015).

Past research has suggested that a single administration of the BMAC technique is a feasible and acceptable procedure in student populations (Holden et al., 2017), adults with PTSD (Panagioti et al., 2012) and individuals experiencing psychosis (Johnson et al., 2013). This preliminary research supports the BMAC as a viable method for inducing and boosting positive affect amongst individuals with psychological difficulties. However, benefits from single session BMAC studies were found immediately following the intervention and not maintained at 2‐week follow‐up. Research has not yet explored the lasting impact of the BMAC when more prolonged socialization and practice is facilitated.

The primary aim of this study was to evaluate the feasibility and acceptability of delivering a brief BMAC intervention to university students experiencing suicidal ideation. A second aim was to explore changes in suicidal ideation and emotional states alongside receipt of the BMAC intervention and after 6 and 12 weeks. Primary hypotheses related to recruitment capability (target of 13 participants, over a 12 month period), completion of outcome measures (>80% of participants), adherence to treatment (>80% of intervention sessions attended), attrition at follow up (<20% of participants) and the suitability and acceptability of the intervention (mean score of >21 indicating acceptability). Secondary hypotheses related to clinical outcomes, with participants expected to experience reduced suicidal ideation and psychological distress at follow‐up.

## 2 METHOD

### 2.1 Design

This study was a single arm pilot study of a brief BMAC intervention in university students experiencing suicidal ideation. This study was approved by a University Research Ethics Committee (2020‐7906‐13023).

### 2.2 Participants

Participants were undergraduate or postgraduate University students in the North West of England who met criteria for experiencing suicidal ideation in the past 4 weeks, determined using the screening question ‘have you had any thoughts about ending your life in the past four weeks?’ It was a requirement that participants were currently accessing a University Counselling Service and were registered with a General Practitioner.

The exclusion criteria were (i) imminent risk to self at the point of recruitment (i.e., if the participant had a plan or intent to end their life in the next 48 hours); (ii) a known formal diagnosis (meeting DSM‐V or ICD‐10 criteria) of bipolar disorder I or II, schizophrenia, schizoaffective disorder, delusional disorder, schizophreniform disorder or other related psychotic disorders; (iii) receiving support from NHS secondary care outpatient or inpatient services; (iv) a known moderate to severe learning disability; (v) a known diagnosis of an Autism Spectrum Disorder; (vi) a known organic cerebral disease/brain injury impacting on receptive and expressive language comprehension; (vii) limited English language ability that would detrimentally impact upon completion of standardized assessments and access to therapy.

### 2.3 Measures

#### 2.3.1 Feasibility outcome measures

Feasibility data related to (i) rates of recruitment; (ii) retention to treatment (number of sessions attended); (iii) completion of between session tasks; (iv) attrition at post treatment and follow up time points; and (v) levels of missing clinical outcome data. Upon session completion, therapists completed a therapy adherence schedule, which recorded session attendance, reason for non‐attendance rates of did not attend (DNA)/could not attend (CNA), duration and content (intervention components delivered).

#### 2.3.2 Acceptability outcome measures

The Client Satisfaction Questionnaire (CSQ‐8; Larson et al., 1979). This was used to measure participant satisfaction upon completion of the intervention. Responses for individual items range from 1–4 and total scores range from 8–32, with higher scores indicating higher satisfaction. The CSQ‐8 is a widely used tool with high internal consistency (coefficient *α* = 0.91) when employed to assess psychotherapy outcomes (Attkisson & Zwick, 1982).

The Acceptability of Intervention Measure, Intervention Appropriateness Measure and Feasibility of Intervention Measure (AIM, IAM, FIM; Weiner et al., 2017). These self‐report measures include 12 items *(on a 5‐point Likert scale*) which rate the participants perceptions of the acceptability, appropriateness, and feasibility of the intervention, which are often considered ‘leading indicators’ of implementation success (Proctor et al., 2011). The AIM, IAM and FIM have demonstrated good structural validity, test–retest reliability and excellent internal consistency (*α* = 0.85–0.91; see Chugani et al., 2020).

#### 2.3.3 Clinical outcome measures

Assessments were completed at baseline, post assessment (6 weeks) and follow‐up (12 weeks).

The Beck Scale for Suicide Ideation (BSS; Beck & Steer, 1991). The BSS is a 21‐item inventory used to rate the intensity of suicidal ideation over the past week. The BSS is a self‐report questionnaire which is widely used in adult populations and has shown strong psychometric properties with university students (e.g., Chioqueta & Stiles, 2006). The BSS has excellent internal consistency (coefficient *α* = 0.94; Mandracchia & Smith, 2015).

Beck Hopelessness Scale (BHS; Beck & Steer, 1988): The BHS is a self‐report measure which comprises 20 true‐false items, scored to indicate the presence of hopelessness and the extent of negative attitudes about the future. The BHS has sound validity and reliability data across samples (e.g., Metalsky & Joiner, 1992). Additionally, it is positively associated with reported suicidal ideation and suicide attempts (Beck & Steer, 1988).

The Patient Health Questionnaire‐9 (PHQ‐9; Kroenke et al., 2001): The PHQ‐9 is a nine item self‐report scale developed to assess the degree of depression present in an individual. The PHQ‐9 is a valid and reliable measure of depression severity and has demonstrated sound psychometric properties in adult primary care samples with high internal consistency (*α* = 0.83; Cameron et al., 2008).

The Generalised Anxiety Disorder assessment‐7 (GAD‐7; Spitzer et al., 2006): The GAD‐7 is a seven item self‐report scale developed to assess the defining symptoms of generalized anxiety disorder. Research has suggested that the GAD‐7 is a valid and reliable screening tool for GAD in primary care settings (Kroenke et al., 2007).

The Positive and Negative Affect Scale (PANAS; Watson et al., 1988): The PANAS is a self‐report scale, which consists of two separate 10‐item scales to measure both positive affect (PA) and negative affect (NA). Watson et al. (1988) rated the internal consistency as high; Cronbach's alpha for the PA and NA scales was 0.89 and 0.88, respectively. Research in non‐clinical samples has also found the PANAS to be a reliable and valid instrument in the assessment of positive and negative affect (Crawford & Henry, 2004).

The Internal State Scale (ISS; Bauer et al., 1991). The ISS is a self‐report scale, comprising 16 items used to measure internal states over the past 24 hours. The ISS consists of four empirically derived subscales: Activation, Well‐Being, Perceived Conflict and the Depression Index. The ISS exhibits good psychometric properties (Paterniti & Bisserbe, 2015), internal consistencies for all ISS scales are reported to be adequate with a coefficient ranging from 0.81 to 0.92 (Bauer et al., 1991).

Perceived Control of Internal States Scale (PCOISS; Pallant, 2000): The PCOISS is a self‐report measure comprising 18 items which measures a persons' perceived control of their thoughts, emotions and bodily sensations. The PCOISS has shown good internal consistency (Cronbach alpha = 0. 92) and reliability (Pallant, 2000).

The BSS (Beck & Steer, 1991) and the ISS (Bauer et al., 1991) were also completed at each intervention session.

### 2.4 Procedure

Individuals were identified and approached by members of staff within university counselling services to participate in the study. Interested individuals received detailed information about the study and a brief telephone screening with the first author to assess eligibility. Eligible participants provided full written consent to participate. Recruitment activities and intervention sessions took place face‐to‐face or online, in accordance with the restrictions in place due to COVID‐19. At baseline, the primary author completed outcome measures, collected demographic information and conducted a risk assessment. At the start of each session, participants completed sessional measures to capture changes in suicidality and affective states across the delivery of the intervention. Outcome measures were collected by the primary author post intervention and at 12‐week follow up. Risk was monitored and managed carefully throughout the study.

### 2.5 Intervention

The BMAC intervention consisted of six one hour individual therapy sessions, delivered over a six week period. This study ran during the global COVID‐19 pandemic. Therefore, most sessions took place via a video platform (e.g., zoom). The intervention sessions included engagement and rapport building, socialization to the BMAC exercise, administration of the BMAC exercise, feedback and debriefing, review of progress and future planning. Each BMAC exercise consisted of a brief relaxation procedure followed by guided imagery of a positive memory, which involved the participant engaging their senses and re‐experiencing positive emotions. The participant was also encouraged to think about their interpretation and appraisal of the situation that resulted in the positive emotion. A copy of the draft intervention manual is available upon request.

Between sessions, participants were encouraged to practice the BMAC exercise. During the first intervention session, participants were provided with a prompt sheet to help identify key focal points within positive memories and an audio recording of the therapist offered prompts to support live practice of the BMAC exercise. Following the completion of the intervention, participants were provided with a therapy blueprint. All intervention sessions were audio recorded. The therapist completed a therapy checklist after every session to assess adherence and fidelity to the manual.

### 2.6 Therapists

A trainee clinical psychologist (primary author) delivered the intervention under the supervision of three qualified clinical psychologists. The trainee received approximately eight hours of clinical supervision including one hour of training in the delivery of the BMAC exercise Discussions focused on the development of the BMAC manual and intervention delivery.

### 2.7 Analysis

Clinical outcome data was analysed with IBM SPSS Statistics (Version 25) and Stata 14.0 (StataCorp., 2015). Wilcoxon signed‐rank tests assessed differences between participants' scores at baseline and post intervention, and at baseline and follow‐up. Summary effect sizes (Cohen's *d*) assessed changes between baseline and post intervention and baseline and follow up (Cohen, 1977), and interpreted using the within‐subject standard deviation (Borenstein et al., 2009). Confidence interval's (CI) for mean differences were also calculated. Individual reliable improvement and deterioration for the BSS was calculated using Jacobson and Truax (1991) Reliable Change Index (RCI).

## 3 RESULTS

Participants were recruited from September 2020 to January 2021. Twelve participants were included within the sample with ages ranging from 18–22 years (mean: 20.5, *SD*: 1.13). Demographic information is available in Table 1.

**TABLE 1:** Demographic information at baseline

Characteristics	Subgroups	*n* (%)
Gender	Female	5 (45%)
Ethnicity	White/White British	8 (72%)
	Asian/Asian other	1 (9%)
	Mixed/multiple ethnic groups	2 (18%)
Living status	Living alone	3 (27%)
	Living with partner	1 (9%)
	Living with friends/students	7 (64%)
Education	Undergraduate study	10 (91%)
	Postgraduate study	1 (9%)
Known diagnoses (self‐report)	No diagnoses	3 (27%)
	Anxiety disorder	2 (18%)
	Depression	4 (36%)
	Personality disorder	1 (9%)
	Other	1 (9%)
Current treatments	Currently receiving therapy	3 (27%)
	Currently prescribed antidepressants	4 (36%)
Past treatment	None	1 (9%)
	Counselling	3 (27%)
	Cognitive Behavioural Therapy	6 (55%)
	Other	1 (9%)

**FIGURE 1:**
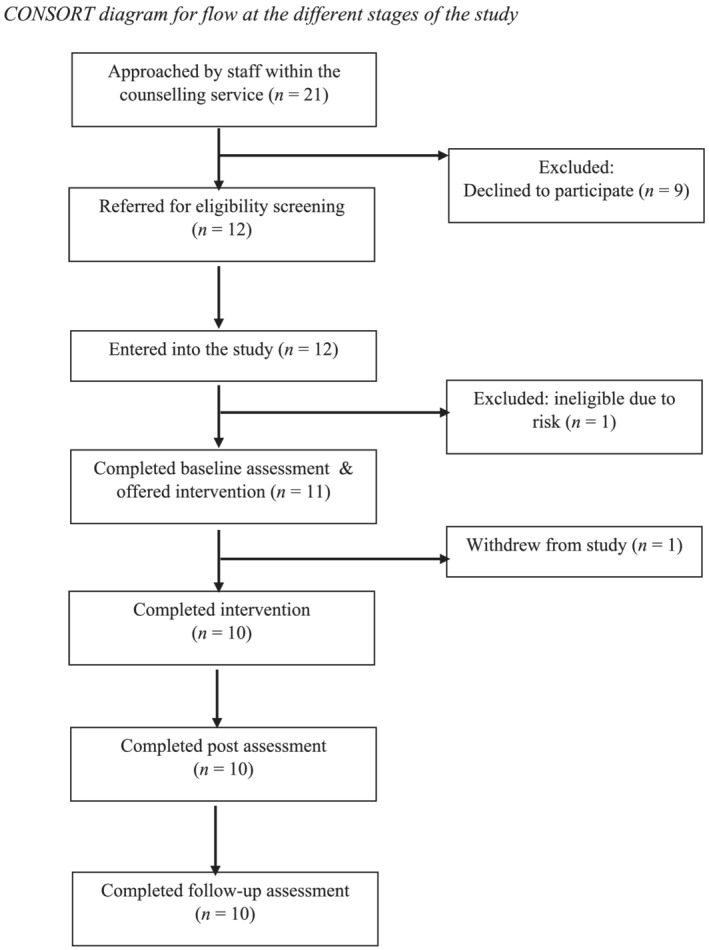
CONSORT diagram for flow at the different stages of the study

The CONSORT diagram in Figure 1 shows the size of the sample at the different stages of the study. Twenty‐one individuals were initially approached to take part with nine declining participation. Feedback from the counselling service suggested that this was due to time constraints and other commitments. All 12 individuals who agreed to be contacted about the study met the eligibility criteria and consented to participate. One participant was excluded during the baseline assessment due to concerns about imminent risk. Eleven participants were offered the BMAC intervention. One participant withdrew from the study after one session, which coincided with the transition to remote delivery, resulting from COVID‐19 restrictions. Thus, 10 participants completed the intervention, all of whom completed the post and follow‐up assessments.

### 3.1 Completion of assessments

One participant failed to attend the post and follow up assessment sessions due to withdrawing from the study, resulting in 93.3% completion of assessments. In participants who attended the baseline, post, and follow‐up assessment sessions, all of the measures (100%) were completed at the three time points.

One participant declined to complete the sessional measures on one occasion due to wanting to prioritize the practice of the BMAC exercise. Otherwise, participants who attended the intervention sessions completed all sessional measures (98%).

### 3.2 Engagement with the intervention

Engagement with the mental imagery intervention was high with participants completing an average of 5.2 sessions (SD = 1.54) out of six. One participant attended one session before withdrawing from the study. Another completed four sessions and two completed five sessions. Reasons for nonattendance were forgetting about the session (*n* = 2), being physically unwell (*n* = 1) and the session being cancelled due to management of risk (*n* = 1). The remaining seven participants attended all six sessions.

### 3.3 Adherence to practice tasks to be completed between session

As shown in Table 2, the number of times that participants practiced the BMAC in between sessions varied considerably. On the whole, participants appeared to practice the BMAC more at the beginning and towards the end of the intervention. The self‐reported reasons for not completing between session practice of the BMAC included: being too busy (*n* = 3), forgetting to practice (*n* = 2), struggling to identify positive experiences or memories (*n* = 3), experiencing distress (*n* = 4), or other unknown reasons (*n* = 1).

**TABLE 2:** Weekly completion of between session tasks

Intervention time point	Weekly percentage of completion	Mean number of BMAC practices *(SD)*	Range (min‐max number of times practiced)
Week 1–2	80%	2.3 (2.41)	0–7
Week 2–3	78%	3.0 (2.24)	0–7
Week 3–4	67%	2.6 (2.79)	0–7
Week 4–5	56%	2.3 (3.46)	0–10
Week 5–6	78%	3.7 (3.57)	0–10

### 3.4 Self‐report measures of acceptability

On the CSQ‐8, the majority of participants rated above 3 (out of 4) on all items, indicating that they were mostly, if not extremely, satisfied with the intervention. Three participants reported lower levels of acceptability, with one participant endorsing multiple items, indicating that this was not the kind of help that they wanted, only a few of their needs had been met, and that the intervention did not help. Two people responded that they would not want to seek the same treatment again. However, overall, a total satisfaction score of 27.2 out of 32 was reported indicating high levels of acceptability across the sample.

The acceptability of intervention measure (AIM) indicated high levels of acceptability, with on average participants scoring 4 or above (out of 5) on all items. One participant reported that the intervention was not appealing. On the intervention appropriateness measure (IAM), the majority of participants agreed that the intervention was appropriate indicating that the intervention was ‘fitting’ (*n* = 7), ‘suitable’ (*n* = 8), ‘applicable’ (*n* = 8) and ‘a good match’ (*n* = 6). No participants disagreed with any of the items on the IAM measure. On the feasibility of intervention measure (FIM), on average, participants agreed that the intervention was ‘doable’, ‘possible’ and ‘implementable’. Two participants disagreed that the intervention was easy to use. Overall, participants rated the acceptability, appropriateness, and feasibility of the intervention as 49.2 out of 60. Acceptability outcome data can be found in Supporting Information S1.

### 3.5 Clinical outcome measures

Summary statistics for primary and secondary outcomes are presented in Table 3. All outcomes improved when comparing baseline to post treatment. The largest effects were observed on the PHQ‐9 (*d* = −1.35) and the BSS (*d* = −1.22). The other outcome measures demonstrated moderate to large effect sizes. More modest changes were observed on the positive subscale of the PANAS (*d* = 0.47) and the depression subscale of the ISS (*d* = −0.33), which demonstrated small effect sizes.

**TABLE 3:** Summary statistics and outcome data for key variables

Variable	Baseline	Post	Follow up	Pre to post		Post mean difference (95% CI)	Pre to follow up		Follow up mean difference (95% CI)
	Mean (SD)	Mean (SD)	Mean (SD)	P	*d*		p	*d*	
BSS	17.0 (3.4)	8.6 (7.1)	8.0 (8.3)	0.009	−1.216	−8.6 (−12.98, −4.22)	0.012	−1.250	−9.2 (−13.76, −4.64)
BHS	12.7 (4.1)	9.7 (5.5)	6.9 (5.7)	0.056	−0.778	−3.1 (−5.57, −0.63)	0.009	−1.117	−5.9 (−9.17, −2.63)
GAD‐7	14.1 (3.4)	11.4 (2.6)	10.5 (4.1)	0.139	−0.514	−2.4 (−5.30, 0.50)	0.199	−0.607	−3.3 (−6.67, 0.07)
PHQ‐9	20.3 (2.4)	15.0 (4.1)	12.6 (6.0)	0.005	−1.348	−5.4 (−7.88, −2.92)	0.011	−1.173	−7.8 (−11.92, −3.68)
PCOISS	49.2 (7.1)	54.8 (3.9)	56.9 (4.0)	0.021	0.705	5.8 (−0.70, 10.90)	0.009	1.025	7.9 (3.12, 12.68)
PANAS subscales									
Positive	20.6 (5.2)	24.1 (6.9)	24.2 (6.4)	0.126	0.474	3.2 (−0.98, 7.38)	0.221	0.449	3.3 (−1.26, 7.86)
Negative	29.7 (4.6)	23.1 (6.2)	25.4 (7.9)	0.016	−0.990	−7.1 (−11.55, −2.65)	0.201	−0.527	−4.8 (−10.45, 0.85)
ISS subscales									
Wellbeing	76.4 (60.5)	124.50 (59.5)	118.5 (53.1)	0.050	0.687	48.5 (4.77, 92.23)	0.113	0.619	42.5 (−0.07, 85.07)
Conflict	147.3 (55.5)	112.0 (58.5)	160.5 (92.4)	0.154	−0.534	−34 (−73.44, 5.44)	0.610	0.133	14.5 (−53.18, 82.18)
Activation	203.2 (133.9)	119.0 (119.4)	112.0 (110.1)	0.028	−0.787	−77.5 (−138.53, −16.47)	0.036	−0.811	−84.5 (−149.05, −19.95)
Depression	103.6 (51.2)	82.50 (44.6)	74.0 (61.0)	0.261	−0.329	−20.5 (−59.16, 18.16)	0.153	−0.512	−29 (−64.12, 6.12)

*Note*: CI = confidence interval, *p* = statistical significance, *d* = effect size (Cohen's *d*), BSS = Beck Scale for Suicidal Ideation, BHS = Beck Hopelessness Scale, GAD‐7 = Generalised Anxiety Disorder Assessment‐7, PHQ‐9 = The Patient Health Questionnaire‐9, PCOISS = Perceived Control of Internal States Scale, PANAS = The Positive and Negative Affect Scale, ISS = Internal State Scale. Cohen's *d* effect size, mean change and 95% CI is based on those with available follow up data only.

The majority of the outcomes also improved between the baseline and 12‐week follow up. Large effects were maintained on the BSS (*d* = −1.25) and the PHQ‐9 (*d* = −1.17). Many of the other outcomes either maintained or increased in effect size from post treatment to 12‐week follow up with the BHS demonstrating the biggest improvement from a moderate effect size (*d* = −0.78) at post treatment to a large effect size (*d* = −1.12) at follow up. Scores on the negative subscale of the PANAS were not maintained; decreasing from a large effect size at post treatment (*d* = −0.99) to a moderate effect size (*d* = −0.53) at follow up. The perceived conflict subscale of the ISS was the only outcome to slightly worsen at follow up in comparison to baseline.

### 3.6 Reliable change

Rates of reliable change were calculated for the BSS at post treatment and 12‐week follow up. This showed a reliable improvement in suicidal ideation for 8 out of the 10 participants retained at follow up. No participants showed a reliable deterioration.

### 3.7 Sessional measures

**FIGURE 2:**
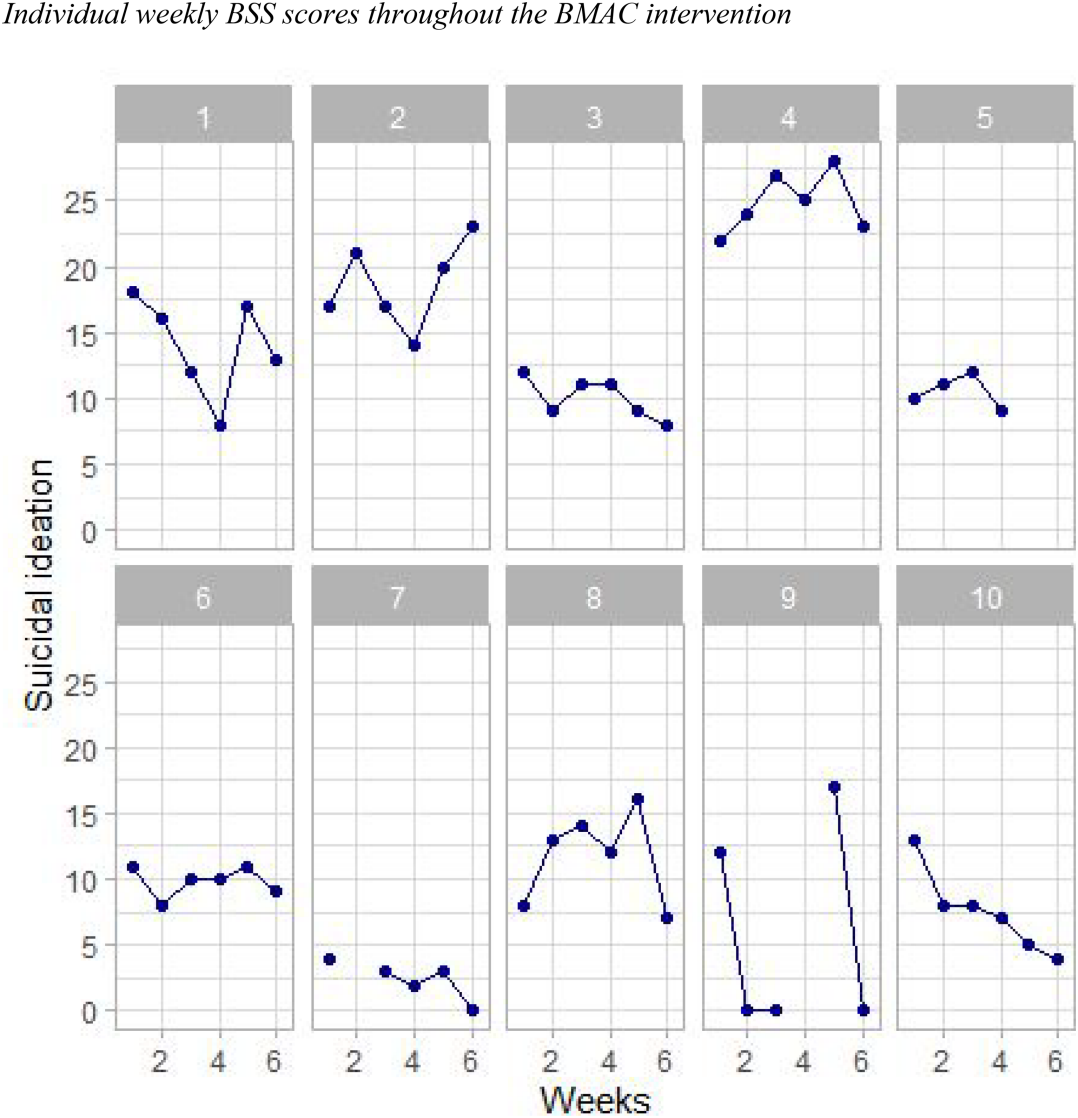
Individual weekly Beck Scale for Suicide Ideation (BSS) scores throughout the Broad‐Minded Affective Coping (BMAC) intervention

Suicidal ideation was assessed on a sessional basis throughout the intervention using the BSS. As shown in Figure 2, there was considerable variation in suicidal ideation within and between participants. The majority (*n* = 8) of participants demonstrated a reduction in suicidal ideation overall. Weekly ISS scores were also variable on all four subscales, but typically indicated an improvement in internal states over time (see Supporting Information S2).

## 4 DISCUSSION

Our results suggest that a brief, six‐session BMAC intervention is feasible for delivery in, and acceptable to, university students experiencing suicidal ideation. This is evidenced by a high rate of participation in the study from referred individuals, excellent adherence to the intervention (average attendance of 5.2 [87%] sessions), and good rates of between session practice. One person withdrew from the study and therefore overall adherence to the assessments was 93.3%. For those who completed the intervention, we observed excellent levels of study retention at post treatment (100%) and follow up (100%). High levels of satisfaction with the intervention were reported by the majority of participants on the CSQ‐8. Most participants rated the intervention as acceptable, appropriate and feasible on the AIM, IAM, and FIM measures. A small minority of participants indicated lower levels of acceptability.

We observed moderate to large improvements across most of the outcome measures following treatment. The greatest reductions were reported for depression on the PHQ‐9 and suicidal ideation on the BSS. We also found a reliable improvement in suicidal ideation for 8 out of 10 participants, indicating a good individual response rate in the reduction of suicidal ideation. Moderate to large improvements across outcome measures were mostly maintained, if not improved, at the follow up assessment. The experience of perceived conflict was the only outcome to slightly worsen at follow up. These preliminary results are promising but should be interpreted with caution; the small sample size and study design mean that effect sizes are likely to be inflated (Parker et al., 2020). Moreover, given the lack of a comparator sample and randomization, these changes cannot be attributed to therapy, as alternative explanations (e.g., effect of monitoring, regression to the mean, concurrent support and treatments) cannot be ruled out. Confirmation in a definitive controlled trial is required to establish if these effects are the results of the intervention and are replicable on a larger scale.

The preliminary findings are consistent with the broaden and build theory (Fredrickson, 2001), which suggests that the stimulation of positive affect can build an individuals' personal resources and increase the odds of successful coping with distress. Teasdale (1988) also theorized that, through increased opportunities to experience positive feelings, there is less opportunity to activate the constellation of negative dysfunctional beliefs and cognitive biases associated with depressed mood. The large increase in the participants perceived control of internal states is also consistent with research suggesting that focusing attention on positive experiences reduces the influence of threat on attentional processes and information processing (Tarrier et al., 2013) and helps to improve attentional control (Tarrier, 2010). Furthermore, contrary to previous research that delivered single time point interventions (Holden et al., 2017; Johnson et al., 2013; Panagioti et al., 2012), this study shows the potential benefit of repeated practice and repetition of the BMAC in facilitating a more sustained improvement in outcomes over time.

Delivery of the brief BMAC intervention is considerably shorter than other treatments designed to target suicidal thinking, such as Cognitive Behavioural Suicide Prevention Therapy (Tarrier et al., 2013). With increased psychological distress observed amongst student populations in recent years, universities have experienced a larger number of students seeking support (Broglia et al., 2018). Indeed, 94% of universities report an increase in demand for counselling services (Thorley, 2017). Therefore, within the context of resource restrictions, this brief BMAC intervention could be a useful and timely treatment option. A possible strength of the BMAC is that it could potentially be taught to a wide range of mental health professionals at different levels of training and skill development, thus increasing the potential for counselling services to adopt this treatment. However, this possibility remains speculative, and research is needed on how readily different professionals could train in this approach. Necessitated by COVID‐19, this study also provides some support for the feasibility of brief psychological interventions delivered online via video conferencing software. Such online platforms are widely accessible and available to many students and such provision may be feasible and acceptable to implement within university counselling services, which requires further investigation.

Regarding methodological limitations, the small sample size reduces statistical power and the precision of estimated effects and the generalizability of the findings (Parker et al., 2020). It is also worth considering the ‘winner's curse’ which highlights the potential for small‐scale studies to provide exaggerated and therefore an unreliable impression of treatment effects (Poldrack, 2019). In an open pilot study, the lack of randomization and the absence of a control group means that the issue of endogeneity cannot be ruled out and it limits the ability to attribute effects to the treatment. Treatment as usual, including access to medication and/or counselling, was also encouraged and not monitored throughout the study, thus limiting the current study's ability to obtain a true reflection of the treatment effect. Furthermore, the assessor was not blind to the presence of treatment, which may have biased the outcomes, including the satisfaction scores. Lastly, this study was designed with a focus on feasibility in line with Medical Research Council (MRC) and National Institute of Health Research (NIHR) guidance (Skivington et al., 2021), and only preliminary investigation of clinical/mechanistic effects could be undertaken. Further investigation of efficacy are now warranted within stronger research designs, such as randomized controlled trials (RCT's).

Despite these limitations, the feasibility and acceptability data and observed effects are encouraging. In the future, research is needed to explore potential mechanisms of change, which may include the development of a sense of agency or control over emotions (Tarrier & Gooding, 2009). Further clarification of the lasting effects of the intervention is also needed; it is unclear whether the maintenance of the observed effects between post intervention and follow up is related to the benefits gained within treatment and ongoing practice of the BMAC. Future studies should consider collecting data on ongoing practice of the BMAC following the end of the intervention. Qualitative work is also required to explore participants' unique experiences of the intervention and to obtain their views on the necessary ingredients that facilitate the successful delivery of the BMAC and any associated barriers and facilitators.

This pilot study shows promising results on the feasibility and acceptability of a brief BMAC intervention. It represents an initial step in exploring a novel intervention to support university students experiencing suicidal ideation.

## Supporting information


**Data S1.** Supplementary file 1: Acceptability outcome data

**Data S2.** Supplementary file 2: Sessional outcome data

## Data Availability

An anonymous copy of the study data is available on request from the corresponding author.
