# Supplementary material for: A positive mental imagery intervention for targeting suicidal ideation in university students: A pilot study
Source: Clin Psychol Psychother. 2022 Feb 13;29(4):1392–402. doi: 10.1002/cpp.2720 (PMC9542303; doi:10.1002/cpp.2720)
Supplement: Supplementary file 1 — Data S1. Supplementary file 1: Acceptability outcome data [file CPP-29-1392-s001.docx]

**Supplementary file 1: Acceptability outcome data**

***Table 1: Total mean and standard deviation scores for individual questions on the Client Satisfaction Questionnaire***

| CSQ questionnaire items | Mean (SD) | Range (1-4) |
| --- | --- | --- |
| 1. How would you rate the quality of the service you received? | 3.9 (0.32) | 3-4 |
| 1. Did you get the kind of service you wanted? | 3.3 (0.67) | 2-4 |
| 1. To what extent has our program met your needs? | 3.1 (0.74) | 2-4 |
| 1. How satisfied were you with the amount of help you received? | 3.4 (0.52) | 3-4 |
| 1. If your friend was in need of similar help, would you recommend our program to him/her? | 3.6 (0.52) | 3-4 |
| 1. Has the service you received helped you deal more effectively with your problems? | 3.2 (0.63) | 2-4 |
| 1. In an overall, general sense, how satisfied are you with the service you have received? | 3.7 (0.48) | 3-4 |
| 1. If you were to seek help again, would you come back to our program? | 3.0 (0.67) | 2-4 |
| Total score | 27.2 (2.82) |  |

***Table 2: Total mean and standard deviation scores for individual questions on the AIM, IAM and FIM.***

| Aim, IAM, FIM questionnaire Items | Mean (SD) | Range (1-5) |
| --- | --- | --- |
| 1. It meets my approval | 4.3 (0.67) | 3-5 |
| 1. Is appealing to me | 4.0 (0.94) | 2-5 |
| 1. I like it | 4.0 (0.67) | 3-5 |
| 1. I welcome it | 4.1 (0.74) | 3-5 |
| 1. It seems fitting | 3.9 (0.74) | 3-5 |
| 1. It seems suitable | 4.1 (0.74) | 3-5 |
| 1. It seems applicable | 4.1 (0.74) | 3-5 |
| 1. It seems like a good match | 3.8 (0.79) | 3-5 |
| 1. It seems implementable | 4.4 (0.70) | 3-5 |
| 1. It seems possible | 4.5 (0.71) | 3-5 |
| 1. It seems doable | 4.4 (0.70) | 3-5 |
| 1. It seems easy to use | 3.6 (1.17) | 2-5 |
| Total score | 49.2 (5.79) |  |
