# Supplementary material for: A positive mental imagery intervention for targeting suicidal ideation in university students: A pilot study
Source: Clin Psychol Psychother. 2022 Feb 13;29(4):1392–402. doi: 10.1002/cpp.2720 (PMC9542303; doi:10.1002/cpp.2720)
Supplement: Supplementary file 2 — Data S2. Supplementary file 2: Sessional outcome data [file CPP-29-1392-s002.docx]

**Supplementary file 2: Sessional outcome data**

***Table 1: Total weekly mean and standard deviation scores for the Internal state subscales***

|  | Internal state subscale means (SD) | | | |
| --- | --- | --- | --- | --- |
| Intervention sessions | Wellbeing | Perceived Conflict | Activation | Depression |
| Week 1 | 95.9 (60.4) | 160.0 (98.3) | 222.3 (128. 8) | 101.8 (38.7) |
| Week 2 | 97.2 (50.4) | 156.7 (62.8) | 225.0 (120.5) | 111.7 (47.4) |
| Week 3 | 84.0 (50.4) | 141.5 (89.8) | 192.5 (120.7) | 93.5 (54.1) |
| Week 4 | 74.4 (33.2) | 142.2 (136.4) | 145.0 (125.5) | 106.7 (40.6) |
| Week 5 | 63.3 (59.4) | 137.8 (89.8) | 156.1 (68.8) | 116.1 (67.8) |
| Week 6 | 125.0 (53.7) | 96.1 (55.6) | 109.4 (49.3) | 73.9 (59.1) |

***Table 2: Total weekly mean and standard deviation scores for the beck scale for suicidal ideation***

| Intervention sessions | BSS total means (SD) |
| --- | --- |
| Week 1 | 14.3 (4.7) |
| Week 2 | 13.4 (6.4) |
| Week 3 | 12.7 (6.7) |
| Week 4 | 11.9 (6.0) |
| Week 5 | 15.0 (7.6) |
| Week 6 | 10.7 (7.8) |
